# Where Do Online Games Fit into the Health Behaviour Ecology of Emerging Adults: A Scoping Review

**DOI:** 10.3390/nu13082895

**Published:** 2021-08-23

**Authors:** David Micallef, Linda Brennan, Lukas Parker, Bruno Schivinski, Michaela Jackson

**Affiliations:** School of Media and Communication, Royal Melbourne Institute of Technology, Melbourne 3000, Australia; linda.brennan@rmit.edu.au (L.B.); lukas.parker@rmit.edu.au (L.P.); bruno.schivinski@rmit.edu.au (B.S.); michaela.jackson@rmit.edu.au (M.J.)

**Keywords:** computer games, behaviour ecological model, social marketing, social network, young people, literature review, eSports

## Abstract

Online video games are a common pastime for emerging adults (EAs). EAs are an age group that is of interest in health communication because habits formed during this life stage can cause or prevent disease later in life. Guided by three research questions, this scoping review identifies the current state of research into socio-ecological influences on physical activity and diet behaviours of EAs. The review also examines the role that online video games play within this behavioural ecology. In total, 112 articles were found that focused on behavioural ecological influences for physical activity and diet behaviour among EAs. Seven of these articles focused on the impact of online video games, although only in conjunction with their influence on physical activity, identifying a gap in understanding the influence of online games on diet. Results show that online video games are currently under-researched in terms of impacts on physical activity and diet despite the prevalence of the use of these games within the EA cohort.

## 1. Introduction

Emerging adulthood, defined as the life stage between the ages of 18 to 25 years, is the peak time of online video game usage [1,2]. In Australia, 67% of emerging adults (EAs) play video games for an average of 89 min per day [1]. As a result of increasingly high-speed internet connectivity, online games—video games played through computer networks—have become a popular form of game play. Online games range from simple single-player games to large virtual worlds featuring thousands of players at once [3]. Despite these considerable usage statistics, online games and associated channels, such as streaming and eSports, are not regularly used in health communication [4]. These channels have, however, become a part of advertising and marketing strategies for technology, lifestyle and food brands wanting to engage EAs [5]. The purpose of this scoping review is to explore the current literature into the health behavioural ecology of EAs and understand the current state of research into online games and their relationship with diet and activity as a part of this ecosystem.

### 1.1. The Importance of Addressing EA Health Behaviour

Chronic health conditions arising from inadequate diets and lack of physical activity are a global health problem [6,7,8]. Emerging adulthood is a critical point in the prevention of future chronic disease because EAs build lifelong habits during this period [9,10,11]. EAs show a drastic reduction in levels of physical activity during emerging adulthood as compared to their adolescence [12,13]. They also eat less fresh fruit and vegetables and consume more energy-dense-nutrition-poor (EDNP) foods such as fast food and sugar-sweetened carbonated beverages [8,14]. The combination of less physical activity, worsening diets, and the importance of this life stage in setting future habits make understanding EAs’ health behaviours important for health promotion professionals globally.

During emerging adulthood, EAs go through major transitions in both their social and physical environments. Socially, EAs make new friends as they enter further education, new living situations, or the workforce, and become less reliant on the networks formed during high school or the influence from parents [10,15]. There are also numerous changes in the physical environment for EAs. For example, EAs often transition to new study environments and/or new workplaces, are likely to move out of their parental home [10,11], and are exposed to new environments with unhealthy food options [16]. EAs are also more likely to move in and out of relationships multiple times during this life stage [10]. The combination of these elements increases the complexity of EAs’ lived experiences and therefore presents challenges for health professionals and communicators seeking to understand these experiences and establish healthful behaviours.

### 1.2. EAs Live in Behavioural Ecologies: Behavioural Ecological Models (BEMs)

Understanding what influences EAs’ daily activities is critical to the development of interventions or campaigns that support their healthy lives. To help understand the complexity of EAs’ lived experiences, behavioural ecological models (BEMs) are useful frameworks because they permit an insight into the myriad of external and internal factors that affect an individual’s behaviour [17]. BEMs take a systems thinking approach to social issues and seek to understand the influences of and connections between actors in an ecological system of behaviours [18]. Systems thinking approaches take into account behavioural ecologies and are beneficial in the dissemination of health information and the implementation of social marketing interventions for health. This is because they aim to consider, account for, and target multiple actors within this ecology [19].

The scoping review used a four-level BEM model [20] to categorize influences on EAs’ behaviours [21]. These levels include: (1) macro-level socio-cultural influences, such as societal mores and traditions; (2) meso-level community influences, such the media and the regulatory environment; (3) meso-level local influences such as communities, workplaces and neighbourhoods; and (4) micro-level individual influences such as family, peers, and individual characteristics, such as pre-dispositions and background

Research into EAs’ behaviours has been focused on behaviour change theories whereby EAs are considered as rational decision makers about lifestyle and health (e.g., Allom, et al. [22]; Hamilton and White [23]). However, the BEM suggests that there are many other bi-directional influences at work across the four levels of the model, as well as positing that social influences predominate due to their proximity to the individual. A BEM framework moves beyond behaviour change theories and helps to understand the context in which EAs are performing the behaviours that we are aiming to change [24,25]. In the context of physical activity and diet, the BEM can help identify the broad range of influences that EAs contend with when living their lives.

EAs engage within their behavioural ecologies differently to other life stages due to the range of instabilities in their lives, from changes to personal relationships and support networks to changes to their physical environments [10]. EAs also engage with digital environments differently to other age groups and have been difficult to reach with health communication [26]. Further, in a rapidly changing digital environment, reaching EAs with the appropriate technology, application, or platform is also quickly changing [27]. For example, EAs have moved away from television in comparison to other age groups, opting for streaming and on-demand services. They also have the highest usage of social media among any age group [28]. The high use of digital media, including video games, is also linked to the health of EAs, with studies showing that increased screen time can have an impact on body mass index and dietary behaviours [29].

### 1.3. Online Games as Part of the Behavioural Ecologies of EAs

Online games have spurred the emergence other activities that complement online game play. These activities include eSports, multi-player online games played as a form of competition between professional players either as individuals or teams [30]. There has also been a similar emergence of game streaming, the broadcast of live video game play in a matter similar to the broadcast of live sports through game streaming platforms such as YouTube or Twitch [3].

Whilst emerging adulthood is the peak for video game usage across all age groups [1,2], little appears to be known about how these games and associated activities exist within the behavioural ecology of EAs’ lived experiences. For example, are games a virtual behavioural ecology? If yes, are the elements of the ecology commensurate with other behavioural ecologies? Do games influence EAs’ engagement across and within their behavioural ecology? If so, how? And importantly, in understanding the influence of games as a form of behavioural ecology, can health communicators leverage online games to enhance health outcomes for EAs?

### 1.4. The Current Study

To answer these questions, we undertook a scoping study using the Arksey and O’Malley [31] framework. Scoping studies are an appropriate methodology when exploring the depth and breadth of a research in a particular field [31]. Scoping studies are particularly necessary when not much is known about the phenomenon in question [29], and where there are no a priori hypotheses to test with more formal meta-analyses or systematic reviews [31]. Scoping reviews aim to map existing research, include a greater range of study designs, and provide a descriptive overview rather than synthesize available research [31,32].

While a significant amount of research is conducted in health settings [7], it is not known what research is being conducted throughout the behavioural ecosystem. As recently outlined by Nagata, et al. [33], important gaps in knowledge should be addressed to advance this important field of research, including examinations of current scholarly literature. Whilst a scoping review into the link between video games and physical health has been conducted by Huard Pelletier et al. [29], to the best of the authors’ knowledge this is the first scoping review to look at online games as part of a broader ecology of physical activity and diet behaviour of EAs.

Considering that, the objective of this scoping review is to identify recent research into the influences that impact physical activity and diet for EAs, specifically with a view to ascertaining the impact of online video games on EAs healthful behaviours. Three research questions guided our scoping review:

- RQ1—To what extent has academic and grey literature explored the behavioural ecological influences of physical activity and diet behaviour of emerging adults?
- RQ2: To what extent has this literature explored online video games as an influence of physical activity and diet behaviour of emerging adults?
- RQ3: Where do online video games fit into the health behaviour ecologies of emerging adults?

## 2. Materials and Methods

For this scoping review we used the five-stage (steps) procedure suggested by Arksey and O’Malley [31]: (i) identify the research question; (ii) identify relevant studies; (iii) select studies using the PRISMA protocol; (iv) chart the data; and (v) collate, summarize and report the results.

In Step 1, the research questions were developed and used to guide the research process. A coding frame for each of the aspects of the BEM was adapted from Parker and Brennan’s work [24,34]. The final coding frame and definitions are provided in Appendix A.

In Step 2, the search terms “emerging adults” and (“physical activity” OR diet) and varying search terms based on the BEM coding frame were used to identify articles on the following six databases: ProQuest (including PsycInfo and Social Science databases), EBSCO (including AMED, Business Source Complete and SPORTDiscus) and Google Scholar. These databases were chosen because they gave a cross section of health and social science literature. Separate searches were also run to identify literature for “computer game*” OR “online game*” OR “video game*” and their variations. The full list of search terms and the results by database are provided in Appendix B. The search was limited to title, abstract, and keywords for the time period of 2010 to 2020 to restrict searches to the most recent literature. In addition, only articles available in English and in full-text form were searched. The initial search identified 146,450 articles. Due to the large number of results, study identification was limited to reviewing the most relevant results from searches. The coding frame was used to identify relevant literature related to the study questions based on a review of the title, keywords, and abstract. A total of 220 articles were identified in this step.

In Step 3, a second review of titles, abstracts, keywords, and methodologies was conducted to identify literature relevant to the study questions and remove titles that did not meet the study relevance criteria. For example, whilst the term “young people” was used as part of the search criteria, a review of methodology was essential at this stage to ensure that studies included participants in the 18–25 age group. Similarly, studies that focused on health but which did not measure physical activity or diet or did not focus on healthy participants were not included. The full-text was not reviewed at this stage. The PRISMA protocol was used as the basis for classifying the studies [35] but was adapted to reflect the methodology. Studies were included if they focused on EAs’ physical activity or diet behaviours or were broader population studies that also included EAs. A total of 111 studies were excluded during this step as they did not include EAs or were not relevant to external influences on physical activity and diet. Therefore, the selection process identified a total of 108 relevant studies. This included the proceedings of two conferences and one thesis due to their relevance to the influence of physical activity and diet behaviour.

**Figure 1:**
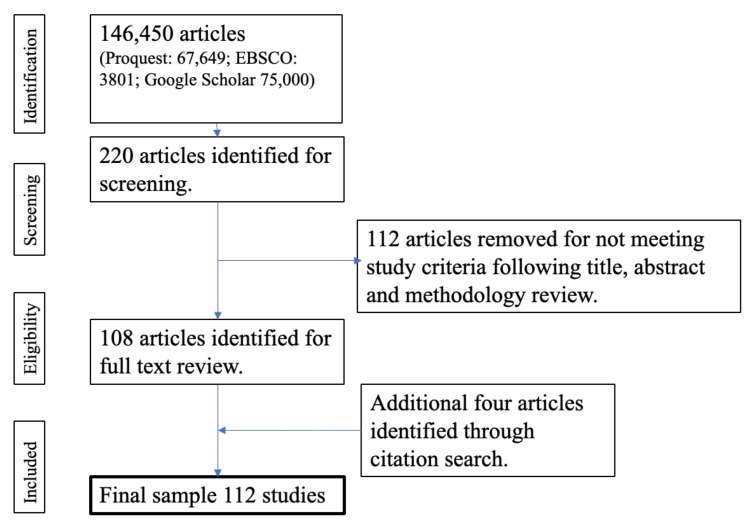
PRISMA flowchart for this study.

A further four articles outside of the original date range were identified through citation searching and were included due to their relevance to the research question, bringing the total to 112. Figure 1 outlines the PRISMA flowchart for the selection of studies.

For the charting of data (Step 4), the full-text was reviewed, and data were manually extracted from the selected studies and imported into Microsoft Excel. Data extracted included the publication details (authors, journal, year of publication, keywords), abstract, study aim, methodology, country of study, population count, and demographics (age and gender or sex where specified). A quality assessment was implemented to explore the validity, results, and relevance of the articles [36]. This was necessary considering the varying age ranges identified that included the study population of 18–25, and the differing definitions of emerging adulthood.

To critically appraise the articles, the hierarchy of evidence framework for assessing healthcare [37] was used as it considers the effectiveness, feasibility, and appropriateness of the study. In addition, articles were assessed based on Brennan et al.’s [21] levels of evidence required for decision making. The combination of the two frameworks meant the study used a well-known quality appraisal system to assess study design combined with a consideration of the direct relevance of the evidence to emerging adulthood. Articles were individually assessed based on both frameworks. No articles were excluded based on the quality appraisal; the investigators used the quality assessment rating in the analysis of findings relevant to EAs, outlined in the results section of this paper.

A coding frame was developed to identify influences on the physical activity and diet behaviour of EAs, grouped by the levels of the BEM. Following charting, studies were analysed (Step 5) through a manual review of the full text, with each influence marked in its relevant category in the database. The final coding frame is provided in Appendix A. Studies often identified multiple influences. In these instances, studies were coded under multiple categories. For example, Amuta, et al. [38]’s qualitative exploration of family, health professional, and peer advice on physical activity was rated under each of these corresponding categories of peer, parents, and health professional. To confirm the analysis, two investigators independently screened a selection (*n* = 10) of included studies against the coding frame. All conflicts were discussed until a joint consensus was reached on the categorization. In addition, the main findings of each study relevant to the influences of physical activity and diet behaviour of EAs were manually entered into the database to allow a textual analysis of findings related to influences. Further analysis was conducted on the participant size of studies, age, the methodological approaches, and the country of origin of the studies.

## 3. Results

**Figure 2:**
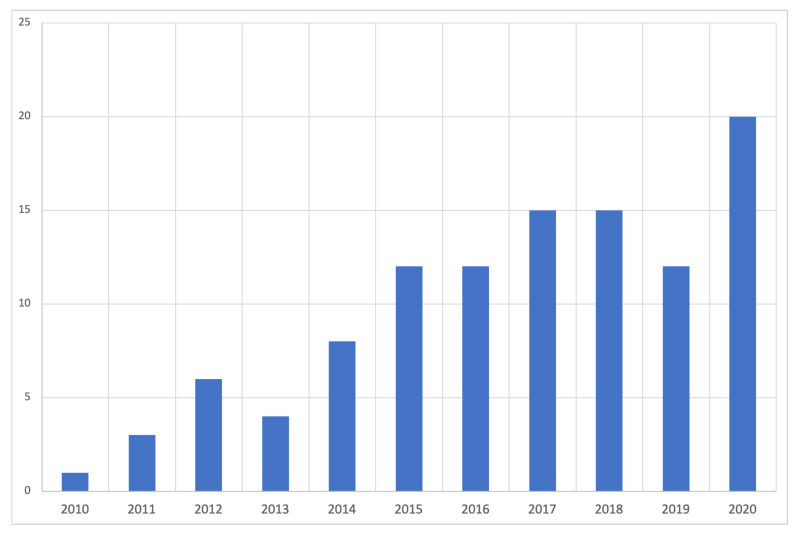
Year of publication analysis for initial search timeline (2010–2020).

The scoping review identified a total of *n* = 112 studies focused on the physical activity and diet behaviour of EAs that attributed behaviours to an external influence. Table 1 tabulates all the studies included in the scoping review, their key characteristics, and their analysis according to the BEM. A supplementary reference list (Supplementary Materials) has been provided that corresponds to the ID numbers listed in Table 1. The review identified 39 studies that looked at both physical activity and diet, 43 studies focusing solely on physical activity, and 30 studies focusing solely on diet. The data highlighted a stronger focus on physical activity, which predominantly focused on measuring levels of exercise in relation to physical activity guidelines. Most studies were published between 2015 and 2020 (*n* = 86; 76.8%), with the most articles in 2020 (*n* = 20; 17.8%). The majority were published in the United States (*n* = 47; 41.9%), followed by Australia (*n* = 20; 17.8%), the United Kingdom (*n* = 12; 10.7%), and Canada (*n* = 9; 8.0%). Figure 2 provides a yearly breakdown of identified studies for the initial search timeline.

Studies included a mixture of qualitative and quantitative methodologies, with quantitative studies including cross-sectional and longitudinal research. Some literature reviews were also included, as well as grey literature (*n* = 3; 2.7%) [39,40,41]. For empirical studies, the study populations ranged from *n* = 18 [42] to *n* = 33,097 [43] and included a range of ages from 13 to 69. Whilst the research question focused on EAs aged 18–25, results identified multiple definitions of the age range for EAs, as well as studies that intersected or included the 18–25 age group. Studies included female, male, and some non-binary participants.

### 3.1. RQ1. Behavioural Ecological Influences on Health

When analysing the frequency of influences based on the group levels of the BEM, individual influences were the focus of a majority of studies (*n* = 151). This was followed by local influences (*n =* 129), community influences (*n* = 96), and socio-cultural influences (*n* = 49). At the single influence level, studies had a strong focus on the university/school experience for EAs (*n* = 72; 64.3%), followed by the influence of peers (*n* = 68; 60.7%), the influence of parents and family (*n* = 35; 31.3%), the impact of social norms (*n* = 31; 27.7%), and the impacts of social media (*n* = 30; 26.8%).

**Figure 3:**
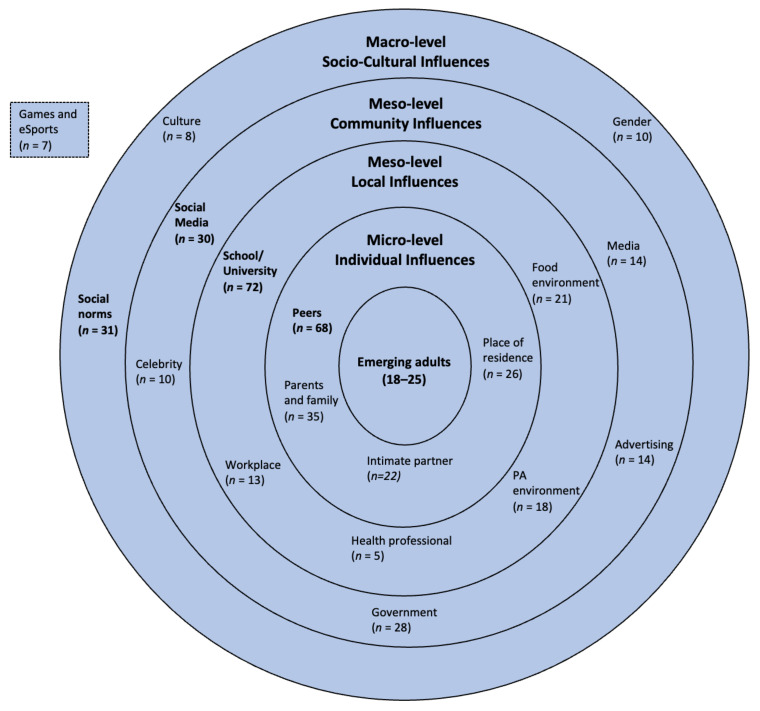
Influences of physical activity and diet behaviour of emerging adults (*n* = number of identified studies). Items in **bold** indicate the highest influence at each level.

Health professionals were the least studied group (*n* = 5; 4.5%), followed by cultural norms (*n* = 8; 7.1%). An overview of the influences identified by the scoping review is presented in Figure 3. It should be noted that most studies (*n* = 105; 93.7%) measured multiple influences, resulting in the total frequency of influences being higher than the total number of studies (*n* = 112).

For socio-cultural influences, research into the influence of physical activity and diet behaviour most often identified the impact that social norms (*n* = 31; 27.7%) have on the behaviour of EAs, followed by gender norms (*n* = 10; 8.9%) and cultural norms (*n* = 8; 7.1%). There was interplay between socio-cultural influences and other influences in the BEM, with all studies that identified the impact of socio-cultural influences also identifying influences at other levels of the model, primarily at the individual level. As an example, Bruening, et al. [44] and Munt et al. [16] found that EAs often feel a societal expectation to eat unhealthily in certain situations, such as birthdays or holiday periods, which are often reinforced by either peers or family members. Similarly, Sogari, et al. [45] identified that EAs often feel a societal pressure to look a certain way and feel an obligation to be seen to be healthy around peers. This was mirrored across other research of socio-cultural influences, highlighting that current research explores this level of influence in conjunction with other influences in EAs behavioural ecology. In addition, the scoping review identified further impacts on EA behaviour based on gender and sexuality. For example, diet and exercise behaviour may be impacted by societal expectations on how much women are supposed to eat or how they are supposed to look [16,46]. Similarly, gay men report a pressure to maintain a certain body weight and shape that drives their eating and exercise routines [43,47].

Research into the influences at the community level focused on social media (*n* = 30; 26.8%) and health promotion by governments (*n* = 28; 25%), as well as the influence of media (*n* = 14; 12.5%), advertising (*n* = 14; 12.5%), and celebrities (*n* = 10; 8.9%). Research often focused on exploring how EAs engage with these influences through digital media. For example, Goodyear, et al. [48] and Sbaffi and Zhao [49] explored how EAs navigate official sources of information from government and health providers, as well as unofficial sources of information from peers, celebrities, and broader community social media posts. There were consistent findings that EAs are sophisticated consumers of health information online [48,50], suffer from exposure to the sophisticated digital presences of the food industry [51,52], and that government health messages are not tailored enough to influence EA behaviour [52,53]. They also actively seek out information and advice on health and food choices from celebrities and micro-celebrities [54].

There were common findings in the dual role that social media plays as an enabler and a barrier to physical activity and diet for EAs [48,53]. For example, food-related social media can encourage unhealthy eating, but can also motivate healthful behaviours when EAs see images of healthy food [55]. In answering the research question, research at this level of the BEM is heavily focused on how EAs interact with popular media and the influencers that reach EAs through these mediums.

Local influences have been heavily studied in relation to physical activity and diet, with a total of 129 local level influences identified (see Figure 3). Research in this field is heavily skewed towards the impact of the study environment or studies that are solely focused on EAs in a university setting, with 72 (64.3%) out of 112 studies focused on the education environment. For example, Diehl and Hilger [56] and Gropper et al. [13] identified the decrease in health outcomes for EAs in the shift from secondary to tertiary education. Laska, et al. [57] and Berg, et al. [58] identified even worse health outcomes for EAs attending two-year versus four-year colleges due to less exposure to campus health services and demographic differences. Studies also identified the impact of interactions with the food environment (*n* = 21; 18.8%) and the physical activity environment (*n* = 18; 16.1%). For example, Kapinos, et al. [59] identified the impact that environmental elements, such as the availability and proximity of food options, as well as the availability of physical activity facilities, has on EAs’ physical activity and diet behaviour. This is further influenced by the wider availability and lower cost of fast-food options [45,60]. EAs living in low-socio-economic status (SES) neighbourhoods experience an increased negative impact, with increased availability of fast food and less access to green spaces, which further limits physical activity [61].

At the local level, studies looking into the impact of the workplace (*n* = 13; 11.6%) focused primarily on the longitudinal effects of being in employment. For example, Bell and Lee [62], Kwan, et al. [63], and Li et al. [8] all identified that EAs in employment fared better than unemployed EAs in terms of physical activity. One study was identified that looked into the workplace factors that could influence healthy or unhealthy behaviours in EAs. Watts, et al. [64] identified the impact that workplace food practices, such as the availability of EDNP food and beverages, can have on EAs’ healthy behaviours. Limited studies were also identified examining the impact of health practitioners (*n* = 5; 4.5%). Studies measuring this influence focused on how EAs access information from health practitioners online, rather than measuring direct interventions through visits with health practitioners.

At the individual level, EAs’ physical activity and diet behaviour was heavily studied, with 151 individual influences identified in the scoping review. The research was concentrated on the influence of peers (*n* = 68; 60.7%), followed by family (*n =* 35; 31.3%), the home environment (*n* = 26; 23.2%), and intimate partners (*n* = 22; 19.6%). Peers were shown to play an increasing role in influencing physical activity and diet behaviour, including how much exercise EAs undertake [65] and the type of food they eat [66]. Peers were found to play a role as motivators [67] and social supports for behaviours [8,68]. However, they can also influence negative behaviours related to diet and exercise [44,45]. The role of parents in influencing these behaviours was less clear. Some studies found that parental influence is less impactful than peers for EAs [66,69,70], while others showed that parents continued to be a stronger influence [8,15,71]. Parental influence was also intertwined with the influence of the home environment, with those still living at home having more access to fresh fruit and vegetables—which are seen as more expensive choices for EAs living away from their parents [44,54,56,72]

The role of intimate partners also has a complex relationship in the influence of physical activity and diet behaviour of EAs. Current intimate partners or the prospect of attracting a romantic or sexual partner can be a strong motivator for exercise and a healthy diet [69,73]. EAs entering relationships, however, have worse health outcomes than their single peers [74], with women faring worse than men [62,75].

### 3.2. RQ2. Extent of Research into the Influence of Online Games on Physical Activity and Diet

When looking at the extent of research into the influence of online games, seven studies (6.3%) were identified that looked at online games’ impact on the health behaviour ecology of EAs. Table 2 provides an outline of these seven studies, their key characteristics, and the intersections with other influences according to the BEM. All seven studies were focused on physical activity and employed a range of study methods including literature reviews (*n* = 2), quantitative studies (*n* = 3), and randomized controlled trials (*n* = 2). No studies were identified that focused on diet. Empirical studies were conducted in the USA (*n* = 2), Canada (*n* = 1), Singapore (*n* = 1), and Hong Kong (*n* = 1), and populations ranged from *n* = 305 [76] to *n* = 1315 [39]. None of the studies looked at the impact of online games on dietary behaviours.

When looking at the focus of the studies, three examined the impact of exergames on physical activity behaviour [76,77,78] and two looked into the impact of the augmented reality game Pokémon Go on physical activity [39,79]. While physical activity is a secondary outcome of augmented reality games like Pokémon Go, exergames incorporate exercising as a primary part of gameplay and include peer-based competitions and interactions with the home environment [78]. EAs, however, do not engage with exergames for the primary purpose of physical activity, with the games often failing to maintain EAs’ interest in the long term [79].

Two studies identified positive impacts of playing video games for EAs. For example, playing sports video games has been found to have an association with participation is real life sports clubs [80]. The longitudinal study found a predictive effect between playing sports video games and increased participation in sports clubs. Non-sport video games were not found to have a similar effect on sport participation. Adachi and Willoughby [80] concluded that EAs’ increased levels of self-esteem from playing sports video games was behind the long-term association with playing sports in real life. EAs playing games online with a range of known and unknown peers can also lead to positive outcomes. For example, Adachi and Willoughby [81] literature review identified that engaging online in cooperative or competitive play can have positive benefits in the way that EAs relate with peers they meet online. This effect was seen regardless of whether EAs were playing violent or non-violent video games.

In answer to the research question, the scoping review identified only a limited set of research into the impact of online games on EAs’ physical activity behaviour, with no results identified on the impact of online games on diet.

### 3.3. RQ3. Where Do Online Games Fit in EAs’ Health Behavioural Ecologies?

When considering where online games fit into EAs’ health behaviour ecology, the scoping review identified intersections at multiple levels of the BEM, including at the individual level (*n* = 3), the local level (*n* = 8), and community influences (*n* = 1).

Peers (*n* = 3) were the only influence identified at the individual level, as online gameplay supports the interaction of EAs with both known peers and peers who EAs meet through online competitive or cooperative gameplay [39,78,81]. The large proportion of studies focused on EAs in tertiary education was mirrored in the studies identified about online games, with four out of the seven studies focused on university students. However, at the local level of the BEM, EAs’ interaction with the physical activity environment (*n* = 4) was also heavily studied. This identified positive links between sports games and sports club participation [80,81] and the exploration of local environments through the augmented reality game Pokémon Go leading to physical activity [39,79].

When comparing the analysis of online game influences with the broader analysis into the body of research reviewed in RQ1, there are a number of gaps highlighted by the results. Out of 17 types of influences identified across this scoping review, four types of influences intersect with online games. None of the research about online games focused on the influence of diet, and no research examined the impact of socio-cultural influences, while only one study included the impact of a community influence [39]. This suggests that while the current results may situate online games at either the individual or local influence level of the BEM, and the extant research is quite limited.

## 4. Discussion

Emerging adulthood is an important stage in establishing lifelong healthy habits. Humans perform their daily lives in behavioural ecologies [53]. Thus, the objective of this scoping review was to identify current research into the influences that impact EAs’ physical activity and diet behaviours using a behavioural ecological model to understand where online games fit into EAs’ behavioural ecologies.

### 4.1. Understanding the Ecology of EAs’ Physical Activity and Diet Behaviour

The findings of this scoping review were consistent with Arnett’s depiction of disruption during this life stage, especially at the individual and local levels of influence [10]. The disruption of social and support networks was a common factor, with newfound peers becoming a major influence on physical activity and diet behaviour [65]. Emerging adulthood is a key stage to assert independence and find a group of peers and a sense of belonging. This makes the influence of peers a critical consideration for research or intervention design to support healthy behaviour.

Influences at other levels of the BEM had ties to individual influences, most often peers, indicating that bi-directional influences are in operation. For example, EAs identified that societal expectations often cause them to eat unhealthily in certain social situations or to feel pressure/expectations to act a certain way (in relation to eating and exercise) [44,45]. It was not discernible whether influences were the result of societal mores or direct influence from peers. The disruption to work, study, and living situations exposes EAs to new physical activity and food environments (i.e., meso-level influences) [59]. However, whether an EA chooses a healthy or an unhealthy option is a complex interplay between access, proximity, cost, and individual influences. For example, connection with sporting groups (local level influence) acts as a protective measure that keeps EAs active and engaged with sport [72,82]. However, group participation can also have negative outcomes on EAs through higher levels of binge-drinking (peer influence) and the advertising, sponsorship, and availability of unhealthy foods at sports facilities (local and community influences) [83]. This interplay highlights that while understanding individual influences is important, working with the full ecology of health behaviour through tools such as the BEM may give greater insights that can support future research and intervention design.

Whilst this review has been focused on identifying research into the influence of online games and where gaming fits in the health ecology of EAs, the scoping review has also identified other gaps in the field. For example, research focused heavily on EAs who are currently in education. Only one study looked into the influence of the workplace [64] and no studies provided any insights into EAs who did not enter education or employment, suggesting gap in both research and interventions for a population that is potentially in greater need. Limited evidence was also found of studies examining the role of health professionals in directly influencing the physical activity and diet behaviours of EAs.

Similarly, eight studies in the scoping review identified an influence based on cultural norms. However, the majority of the cultural influences identified were secondary to the main purpose of the study. For example, Baiocchi-Wagner and Talley [84] and Guntzviller, et al. [85] studies measuring the role of parental influence found that EAs from cultures that traditionally place a lot of value in the role of elders had stronger levels of parental influence. Uijtdewilligen et al. [75] hinted at cultural differences in the research population but did not study cultural influences directly. Conversely, Walker et al.’s study into the health of Indigenous Australian EAs took a direct approach at understanding the cultural influences that impacted Indigenous Australians’ health [42]. There is further opportunity to explore the role that cultural norms continue to play in EAs’ behaviours, especially through the shift between parental/family and peer influences during this life stage.

### 4.2. Understanding the Influence of Online Games

Considering the high level of usage of online games for EAs, there is substantial potential to study the impacts of online games on EAs’ physical activity and diet behaviour.

There are a number of intersections between online games and influences at different levels of the BEM, such as the intersection of physical activity environments [39,80] with peer relationships [81]. There are other intersections that were not explored. For example, many online games place the user in a vast and immersive virtual world where food and physical activity behaviours are a common part of their character’s and avatar’s experience [86]. However, no research was identified on the impact of these behaviours in virtual worlds on EAs’ physical activity and diet behaviours. In contrast, in-game health behaviours have been shown to have an impact on real-world behaviour. For example, Cranwell, et al. [87] study on the impact of tobacco and alcohol use in video games showed a correlation between exposure to these virtual behaviours and actual use. Furthermore, no relationships were found on the influence of food advertising in online games, even though the use of online games, eSports, and game streaming platforms is now a common part of the marketing strategies for brands wanting to engage with EAs [5]. Intersections were also not found between online games and socio-cultural influences, even though the broader scoping review found other mediums such as social media, mainstream or traditional media, and advertising intersect with social, cultural, and gender norms in relation to physical activity and diet.

There is no apparent research on game-specific microcelebrities and their influence on physical activity and diet behaviour for EAs, despite the growth of this sector in recent years [5]. Microcelebrities are social media users who amass large followings through the personal content that they share [4]. Game-specific microcelebrities use streaming platforms such as Twitch to stream their gameplay live to audiences that can reach into the millions. Microcelebrities have been shown to have a level of influence with their audience that can mimic the influence of peers [4]. Lee et al. [4] study identified that mental health disclosures by microcelebrities on Twitch had positive impacts on the mental health help-seeking behaviour of their audience. They identified a lack of research into how powerful influencer channels may be used to engage audiences with positive health messages. Research identified in this scoping review focused on the influence of celebrities and social media influencers. Further investigation into game-specific microcelebrities may identify a new channel of reaching EAs, especially men, who are under-represented in microcelebrity channel posts about physical activity, nutrition, and diet [88].

Online games provide EAs the opportunity to engage with their peers in virtual worlds, whether they be known peers that they also engage with offline or new peers that they know only online. Through the competitive and cooperative nature of online games, players form teams and social groups to engage with the games. There is an opportunity for future research to explore whether there is a potential to leverage these virtual peer relationships to encourage healthful dietary behaviours and physical activity. Recent work by Parkinson, et al. [89] identifies a framework for the use of online third places to support the improvement of wellbeing. With an increasing number of massively multiplayer online games (MMOGs) allowing the establishment of branded, interactive services, there is an opportunity for future research to explore the potential for these spaces to move beyond information channels and engage EAs in healthful behaviours.

### 4.3. Limitations

This scoping review describes and analyses the current body of knowledge on the physical activity and diet behaviour influences of EAs. The review identified that there is a large amount of research, and while it was carefully conducted, the method of the study meant it was not feasible to go through all the research in this field. There may be other relevant studies that may have not been included because they did not fit the search strategy or selection criteria of this review. Initial database searches identified a large number of articles (*n* = 146,450) however, the methodology did not allow for an analysis of duplicates in searches. Much research is not written in English and was therefore not included. The research was further restricted to the years 2010 to 2020 to capture the most up to date insights concerning EAs, especially as online games is a relatively recent phenomenon enabled by new technologies. The studies on online games and digital media usage were also based on the collection of data prior to 2020. The impact of the COVID-19 pandemic on the use of digital environments has been well-reported, and current EA usage statistics are likely to be much higher than those reported in this scoping review. However, given that the rapid rise in COVID-19-related research was likely to skew the results, the research team opted to exclude 2021 from the review.

## 5. Conclusions

The importance of emerging adulthood in the prevention of future disease has resulted in a large focus of research on understanding EAs’ behaviours. However, to the best of our knowledge, this is the first scoping review that uses a behavioural ecological model to identify key influences on EAs’ lived experiences. This scoping review identified a large body of relevant research that seeks to understand different influences and the impact of these influences on physical activity and diet behaviour. While research has explored a number of key areas of the behavioural ecology, research into the influence of online games on the healthy behaviour of EAs is limited. Considering the high usage of this medium by EAs, this suggests an opportunity for further investigation into the intersection between online games and health behaviours such as diet and activity, and how these may be engaged for the purpose of health communication. This review has identified a number of opportunities for future research, especially in relation to the application of online games to education about health and diet.

## Figures and Tables

**Table 1:** Characteristics of scoping review studies—all studies.

SRID	Year	Participants/ (Studies)	Country	Age	Behaviour		Individual Influences				Local Influences					Community Influences					Socio-Cultural Influences			OG
					PA	Diet	Peer	Partner	Home	Parents	Work	Edu	FE	HP	PA Env	Social Media	Cel	Gov	Media	Adv	Cult Norm	Social Norms	Gender	
1	2017	1132	Canada	17–25	1	0	0	0	0	0	0	1	0	0	1	0	0	0	0	0	0	0	0	1
2	2017	Not stated	N/A	N/A	1	0	1	0	0	0	0	0	0	0	1	0	0	0	0	0	0	0	0	1
3	2016	58	Saudi Arabia	18–24	1	0	0	0	0	0	0	1	0	0	0	1	0	0	0	0	0	0	0	0
4	2014	35	Australia	19.46 (mean)	0	1	0	0	1	0	0	1	1	0	0	0	0	0	0	1	0	0	0	0
5	2016	101	Australia	17–54	1	0	1	0	0	0	0	1	0	0	0	0	0	0	0	0	0	1	0	0
6	2017	319	USA	18–24	1	0	1	0	0	1	0	1	0	1	0	0	0	0	0	0	0	0	0	0
7	2015	61	Australia	18–24	1	1	1	1	0	1	0	0	0	0	0	1	0	0	0	0	0	1	0	0
8	2013	866	USA	18–27	1	1	0	0	0	1	0	0	0	0	0	0	0	0	0	0	1	0	0	0
9	2010	3610	Australia	18–46	1	1	1	0	0	1	0	0	0	0	0	0	0	0	0	0	0	1	0	0
10	2020	N/A	Australia	N/A	0	1	0	0	0	0	0	0	0	0	0	1	1	1	0	1	0	0	0	0
11	2014	129	USA	Not stated	1	0	1	0	0	0	0	1	0	0	0	0	0	0	0	0	0	1	0	0
12	2018	1043	USA	18–24	1	0	1	0	0	1	0	1	0	0	0	0	0	0	0	0	0	0	0	0
13	2005	8545	Australia	18–27	1	0	0	1	1	0	1	1	0	0	0	0	0	0	0	0	0	0	1	0
14	2013	2265	USA	18–25	1	1	1	0	0	0	0	1	1	0	0	0	0	1	0	0	0	0	0	0
15	2018	19	Scotland	18–25	1	1	1	0	0	1	0	0	0	0	0	1	0	1	1	0	0	0	0	0
16	2017	98	USA	17–35	1	0	0	1	0	0	0	1	0	0	0	0	0	0	0	0	0	0	0	0
17	2004	145	Canada	17–19	1	0	0	0	0	0	0	1	0	0	0	0	0	0	0	0	0	0	0	0
18	2020	195	Australia	18–24	0	1	1	0	0	0	0	0	0	0	0	1	0	1	1	1	0	1	0	0
19	2020	195	Australia	18–24	0	1	0	0	0	0	0	0	0	0	0	1	1	0	0	0	0	0	0	0
20	2014	60	Canada	17–19	1	1	1	0	1	0	0	1	1	0	0	0	0	0	0	0	0	0	0	0
21	2017	52	USA	18–24	1	1	1	0	1	0	0	1	1	0	0	0	0	0	0	0	0	1	0	0
22	2020	11,462	UK	16–24	1	0	1	0	0	0	0	1	0	0	0	0	0	0	0	0	0	0	0	0
23	2020	780	USA	21–50	1	1	1	0	0	0	0	0	0	0	1	0	0	0	0	0	0	0	0	0
24	2020	242	Australia	18–30	0	1	1	0	0	0	0	0	0	0	1	0	0	0	0	1	0	1	0	0
25	2020	608	Spain	19–23	1	0	1	1	0	1	0	1	0	0	1	0	0	0	0	0	0	0	0	0
26	2018	490	Spain	20–29	1	1	1	0	0	0	0	1	0	0	0	0	0	0	0	0	0	0	0	0
27	2019	30 (studies)	N/A	N/A	1	0	1	0	0	0	0	0	0	0	0	0	0	0	0	0	0	0	0	1
28	2020	317	Netherlands	18–24	1	0	0	0	0	0	0	1	0	0	0	0	0	0	0	0	0	0	0	0
29	2018	47	USA	17–19	0	1	0	0	0	1	0	0	0	0	0	0	0	0	0	0	0	0	0	0
30	2019	67 (studies)	UK	13–30	1	0	0	0	0	0	0	0	0	0	0	0	0	0	0	0	0	0	0	0
31	2016	689	Germany	18–24	1	0	1	1	1	1	0	1	0	0	0	0	0	0	0	0	0	0	0	0
32	2019	330	USA	18–24	1	0	1	0	0	0	0	1	0	0	0	1	0	0	0	0	0	0	0	0
33	2015	42 (studies)	Worldwide	13–19	1	1	1	0	0	1	0	0	0	0	0	0	0	0	0	0	0	0	0	0
34	2013	34	Scotland	14–18	1	1	1	0	0	0	0	0	0	0	0	1	0	1	0	0	0	0	0	0
35	2011	10 (studies)	USA Australia	13–19	0	1	1	0	0	0	0	0	0	0	0	0	0	0	0	0	0	1	1	0
36	2015	N/A	N/A	18– 24	1	1	0	0	0	0	0	0	0	0	0	1	1	1	1	1	0	1	0	0
37	2018	30	USA	18–24	1	0	1	0	0	1	0	0	0	0	0	0	0	0	0	0	0	0	0	0
38	2014	50	England	19–26	1	1	0	0	0	0	0	0	1	0	0	0	0	1	1	0	0	0	0	0
38	2018	1600	UK	13–19	1	1	0	0	0	0	0	1	0	0	0	1	1	1	1	1	0	1	0	0
40	2019	1296	UK	13–18	1	1	1	0	0	0	0	1	0	0	0	1	1	1	1	1	0	1	0	0
41	2013	1201	USA	19–30	0	1	1	0	1	1	0	1	1	1	0	0	0	0	0	0	0	0	0	0
42	2020	107 (studies)	USA	N/A	1	0	0	1	1	0	1	1	0	0	0	0	0	0	0	0	0	0	0	0
43	2017	196	USA	18–28	1	0	0	0	0	1	0	1	0	0	0	0	0	0	0	0	1	0	0	0
44	2018	60	Saudi Arabia	18–29	0	1	0	0	1	0	0	1	0	0	0	1	0	1	0	0	0	0	0	0
45	2015	200	Finland	18–50	1	0	1	0	0	0	0	0	0	0	0	0	0	0	0	0	0	1	0	0
46	2016	40	USA	18–30	1	1	1	1	0	1	0	1	0	0	0	0	0	0	0	0	0	0	0	0
47	2016	N/A	N/A	N/A	1	0	1	1	0	0	0	0	0	0	0	0	0	0	0	1	0	1	1	0
48	2015	620	Australia	18–30	1	1	1	1	1	1	1	0	1	0	0	0	0	0	0	0	0	0	0	0
49	2018	N/A	N/A	N/A	1	1	1	1	0	1	1	1	1	1	1	0	0	1	1	0	1	1	0	0
50	2019	235	USA	18–24	0	1	1	0	0	0	0	1	0	0	0	0	0	1	1	0	0	1	0	0
51	2020	22 (studies)	USA	18–30	0	1	1	0	0	0	0	0	0	0	0	1	1	1	0	0	0	0	0	0
52	2014	3825	USA	Not stated	0	1	0	0	1	0	0	1	1	0	0	0	0	0	0	0	0	0	0	0
53	2015	216	Korea	17–29	1	0	1	0	0	0	0	1	0	0	0	0	0	0	0	0	0	0	0	0
54	2012	89	USA	16.3 (mean)	0	1	1	0	0	1	0	1	0	0	0	0	0	0	0	1	0	0	0	0
55	2014	480	USA	18–24	1	0	1	1	0	1	0	1	0	0	0	0	0	0	0	0	0	1	0	0
56	2018	67	Canada	17–24	1	1	1	0	0	0	0	1	0	0	0	0	0	0	0	0	0	1	0	0
57	2018	23 (studies)	Australia	18–24	0	1	1	0	0	0	0	0	0	0	0	1	0	1	0	0	0	0	0	0
58	2019	1315	USA	15–65	1	0	1	0	0	0	0	0	0	0	1	1	0	0	0	0	0	0	0	1
59	2017	499	Germany	20–25	0	1	1	0	0	0	0	1	0	0	0	1	1	0	0	0	0	1	0	0
60	2012	640	Canada	24–27	1	0	0	0	0	0	1	1	0	0	0	0	0	0	0	0	0	0	0	0
61	2012	312	USA	18–40	1	1	0	0	0	0	0	1	0	0	0	1	0	0	0	0	0	0	0	0
62	2019	49	Australia	18–25	1	1	1	0	1	1	0	1	1	0	0	1	1	1	0	1	0	1	1	0
63	2017	435	USA	18–25	1	1	0	0	0	1	0	1	0	0	0	0	0	0	0	0	0	0	0	0
64	2018	1120	USA	14–31	1	1	1	1	1	1	0	0	1	0	1	0	0	0	0	0	0	0	0	0
65	2012	1130	USA	14–31	0	1	1	1	1	1	0	0	0	0	0	0	0	0	0	0	0	0	0	0
66	2016	441	USA	18–35	1	1	0	0	0	0	0	1	1	0	0	1	0	1	0	0	0	0	0	0
67	2015	33,097	USA	18–29	1	1	0	0	0	0	0	1	0	0	0	0	0	0	0	0	0	0	1	0
68	1999	2729	Australia	15–76	1	0	1	0	0	1	1	1	1	0	1	0	0	0	0	0	0	0	0	0
69	2020	N/A	N/A	N/A	1	0	0	0	0	0	0	0	0	0	0	0	0	1	1	0	0	0	0	0
70	2016	322	Singapore	10–18	1	0	0	0	0	0	0	1	0	0	0	0	0	0	0	0	0	0	0	1
71	2016	2795	UK	16–20	1	0	1	0	1	1	1	1	0	0	0	0	0	0	0	0	0	0	0	0
72	2015	109	USA	18+	0	1	1	0	0	0	0	1	0	0	0	0	0	1	1	0	0	1	0	0
73	2020	55	Sweden	18–25	1	0	1	0	0	0	0	0	0	0	1	0	0	0	0	0	0	0	0	0
74	2016	12,164	USA	15–19	1	1	0	0	0	0	0	0	1	0	1	0	0	0	0	0	0	0	1	0
75	2020	30	Australia	16–25	1	0	1	0	0	1	0	0	0	1	0	1	0	1	0	0	0	0	0	0
76	2011	104	USA	18–40	1	0	0	0	0	0	0	1	0	0	0	1	0	0	0	0	0	0	0	0
77	2016	188	Canada	17–55	0	1	1	0	0	1	0	1	0	1	0	1	0	1	1	1	0	0	0	0
78	2017	857	Canada	18–24	0	1	1	1	1	1	0	1	1	0	0	0	0	1	0	0	0	0	0	0
79	2017	111	USA	18–22	0	1	1	0	0	0	0	1	0	0	0	1	0	0	0	0	1	1	0	0
80	2020	59	Australia	21–29	0	1	0	0	1	1	0	0	0	0	0	0	0	1	0	1	0	1	0	0
81	2020	166	Australia	18–24	1	1	1	0	1	1	0	0	1	0	0	1	0	1	1	1	0	1	0	0
82	2017	34 (studies)	Australia	18–24	0	1	1	1	1	1	0	1	1	0	0	1	0	0	0	0	1	1	1	0
83	2020	305	USA	18–37	1	0	0	0	0	0	0	1	0	0	0	0	0	0	0	0	0	0	0	1
84	2017	2397	Australia	18–34	0	1	0	0	1	0	0	0	0	0	0	0	0	0	0	0	0	0	0	0
85	2019	84	USA	19–34	1	0	1	0	0	0	0	1	0	0	0	0	0	0	0	0	0	0	0	0
86	2018	404	USA	21–35	1	0	0	1	1	0	1	1	0	0	0	0	0	0	0	0	0	0	0	0
87	2014	1000	USA	18–29	0	1	1	1	0	1	0	1	0	0	0	0	0	0	0	0	0	1	0	0
88	2012	1201	USA	17–24+	0	1	0	1	1	0	1	1	0	0	0	0	0	0	0	0	0	0	1	0
89	2008	310	Australia	18–24	0	1	0	0	1	0	1	0	0	0	0	0	0	0	0	0	1	0	0	0
90	2015	41 (studies)	USA	N/A	1	1	0	0	0	0	0	1	0	0	0	0	0	0	0	0	0	0	0	0
91	2012	1313	Scotland	18–25	1	0	1	1	0	1	0	1	0	0	0	0	0	1	0	0	0	1	0	0
92	2014	227	USA	16–22	1	0	1	0	0	1	0	1	0	0	0	0	0	0	0	0	0	0	0	0
93	2015	N/A	N/A	N/A	0	1	1	0	0	0	0	0	0	0	0	0	0	1	0	0	0	1	0	0
94	2011	172	UK	18–24	0	1	1	0	0	0	0	1	0	0	0	0	0	0	0	0	0	1	0	0
95	2019	11,125 (studies)	Worldwide	18–30	0	1	1	0	0	0	0	0	0	0	0	1	1	1	0	0	0	0	0	0
96	2020	291	USA	25.5 (mean)	1	1	0	0	0	0	0	1	0	0	0	1	0	1	1	0	0	0	0	0
97	2017	819	Canada	17–21	1	0	1	0	1	1	0	1	0	0	0	0	0	0	0	0	0	0	0	0
98	2019	21	UK	19–21	0	1	1	0	0	0	0	1	0	0	0	1	0	0	0	0	0	1	0	0
99	2018	35	USA	19–22	1	1	1	0	1	1	0	1	0	0	1	0	0	0	0	1	0	1	0	0
100	2018	185	USA	N/A	1	1	1	0	0	0	0	1	0	0	0	0	0	0	0	0	0	0	0	0
101	2019	102	USA	18–25	1	1	0	0	0	0	0	1	0	0	0	0	1	0	1	0	0	0	0	0
102	2017	N/A	UK	N/A	1	1	0	0	1	0	0	0	1	0	1	0	0	0	0	0	0	0	0	0
103	2015	11,695	Australia	22–27	1	0	0	1	0	0	1	1	0	0	0	0	0	0	0	0	1	0	1	0
104	2019	2829	Netherlands	18–45	1	0	0	1	0	0	1	1	0	0	1	0	0	0	0	0	0	0	0	0
105	2016	30	USA	18–30	1	1	1	1	0	0	0	1	1	0	0	0	0	1	0	0	0	1	1	0
106	2015	34	USA	18–25	1	1	1	0	0	0	0	1	0	0	0	1	0	0	0	0	0	0	0	0
107	2020	18	Australia	17–24	1	1	1	0	0	0	0	0	0	0	0	1	0	0	0	0	1	0	0	0
108	2018	48	Canada	18–30	1	1	0	0	1	0	0	1	1	0	1	0	0	0	0	0	0	0	0	0
109	2016	2287	USA	18–27	1	1	1	0	0	0	1	0	1	0	1	0	0	0	0	0	0	0	0	0
110	2017	644	Hong Kong	18–24	1	0	0	0	0	0	0	1	0	0	1	0	0	0	0	0	0	0	0	1
111	2020	31,067	USA	22 (mean)	1	0	0	0	0	0	0	1	0	0	0	0	0	0	0	0	0	0	0	0
112	2020	229	USA	17–20	1	1	0	0	0	0	0	1	0	0	1	0	0	0	0	0	0	0	0	0

Abbreviations: 1 = Behaviour or influence detected as per coding frame; 0 = Behaviour or influence not detected; SRID = Corresponding paper in Supplementary Reference List; PA = physical activity; Home = Home environment; Edu = Education; FE = Food environment; HP = Health practitioner; PA env = PA environment; Cel = Celebrity/Microcelebrity; Gov = Government/health promotion; Adv = Advertising; Cult norm = Cultural norms; Gender = Gender norms; OG = Online game and eSports; N/A: Element does not apply to the study; Not stated: Element not indicated in study.

**Table 2:** Characteristics of scoping review studies—online game studies only.

SRID	Year	No of Participants/(Studies)	Country	Age Group	Behaviour		Individual Influences				Local Influences					Community Influences					Socio-Cultural Influences			OG
					PA	Diet	Peer	Partner	Home	Parents	Work	Edu	FE	HP	PA env	Social Media	Cel	Gov	Media	Adv	Cult Norm	Social Norm	Gender	
70	2016	322	Singapore	10–18	1	0	0	0	0	0	0	1	0	0	0	0	0	0	0	0	0	0	0	1
1	2017	1132	Canada	17–25	1	0	0	0	0	0	0	1	0	0	1	0	0	0	0	0	0	0	0	1
2	2017	Not stated	N/A	N/A	1	0	1	0	0	0	0	0	0	0	1	0	0	0	0	0	0	0	0	1
110	2017	644	Hong Kong	18–24	1	0	0	0	0	0	0	1	0	0	1	0	0	0	0	0	0	0	0	1
27	2019	30 (studies)	N/A	N/A	1	0	1	0	0	0	0	0	0	0	0	0	0	0	0	0	0	0	0	1
58	2019	1315	USA	15–65	1	0	1	0	0	0	0	0	0	0	1	1	0	0	0	0	0	0	0	1
83	2020	305	USA	18–37	1	0	0	0	0	0	0	1	0	0	0	0	0	0	0	0	0	0	0	1
**TOTALS**					7	0	3	0	0	0	0	4	0	0	4	1	0	0	0	0	0	0	0	7

Abbreviations: 1 = Behaviour or influence detected as per coding frame; 0 = Behaviour or influence not detected; PA = physical activity; Home = Home environment; Edu = Education; FE = Food environment; HP = Health practitioner; PA env = PA environment; Cel = Celebrity/Microcelebrity; Gov = Government/health promotion; Adv = Advertising; Cult norm = Cultural norms; Gender = Gender norms; OG = Online game and eSports; N/A: Element does not apply to the study; Not stated: Element not indicated in study.

## Supplementary Materials

The following are available online at https://www.mdpi.com/article/10.3390/nu13082895/s1, Supplementary Reference List – articles included in scoping review.

## Appendix A. Coding Frame

**Table A1:** Coding frame to categorise scoping review studies according to behaviour and the BEM.

Category	Definition	Reference
**Behaviour**		
Physical Activity	Studies focused on the measurement of movement to expend energy (physical activity) or planned activity to improve or maintain physical fitness (exercise)	[90]
Diet	Studies measured the types of food that a person eats (diet) and/or the consumption of food based on dietary guidelines (nutrition)	
**Individual influences**		
Peer	Studies identified the influence of a person in the same social group and/or demographic with which the person regularly interacts	
Partner	Studies identified the influence of a person the individual is romantically or sexually involved with, or the influence of their behaviour based on a desire to attract a future romantic or sexual partner.	
Home environment	Studies identified the influence exerted by the conditions of the environment in which a person lives, such as availability of cooking facilities, or ease of access to healthy or unhealthy food inside the home.	
Parents and family	Studies identified the influence of the parent/child relationship or of relatives	
**Local influences**		
Workplace	Identifies the influence of a place of employment	
School/university	Study identifies the impact of the study environment, or the study is solely based on students in a school or tertiary education setting	
Food environment	Study identified the influence of local infrastructure that exposes individuals to healthy or unhealthy food choices such as the availability of fast-food restaurants.	[59]
Health professional	Study mentions the influence of a doctor or a health practitioner	
Physical activity environment	Study identifies the influence of local and community spaces that encourage physical activity, such as parks, walking tracks and the sporting facilities and clubs	[59]
**Community influences**		
Social media	Identified the influence of any web-based communication channel, most commonly well-known platforms like Facebook, Instagram and YouTube.	[91]
Celebrities/micro-celebrities	Study identified the influence of a mainstream public person (celebrity) or an individual who has gained a large following through social media (microcelebrity) in impacting behaviour.	[4,92]
Government/health promotion	Study identified the influence of health information and health interventions, most commonly issued by government, health promotion or related agencies	
Media	The influence by popular culture through traditional media such as film and television.	
Advertising	Study identified the influence of the advertising of food and drink or the advertising of social norms relating to eating, drinking and activity	[93]
**Socio-cultural influences**		
Culture	Study identifies the influence of behaviour expectations based on belonging to an ethnic or cultural group	
Social norms	Study identifies the influence of behaviour based on societal expectations (not directly related to ethnic groups or gender norms)	[94]
Gender	Study identified the influence of behaviour based on gender norms	
Online games and eSports		
Games and eSports	The influence of computer games and organized online games activities and competitions (esports)	[5]

## Appendix B. Search Terms

**Table A2:** Scoping review search terms and result totals.

Database	Search Level 1	Search Level 2 (AND)	Search Level 3 (AND)	Date Range	Results	
ProQuest	“physical activity” OR exercise OR fitness OR “physical exercise” OR diet OR nutrition *n* = 974,915	“young adult*” OR “emerging adult*” OR “young people” *n* = 74,937	influence* OR “social influences” OR peer OR parent OR partner OR environment OR work* OR school OR university OR “social media” OR advertising OR “health communication” OR “video game*” OR “online game*” OR “computer game*” OR government OR “social norm” OR celebrity OR microcelebrity OR “physical activity environment” OR obesogenic OR “food environment”	2010–2020	67,649	
ProQuest	“physical activity” OR exercise OR fitness OR “physical exercise” OR diet OR nutrition *n =* 974,915	“young adult*” OR “emerging adult*” OR “young people” *n =* 74,937	“video game*” OR “online game*” OR “computer game*”	2010–2020	381	*not counted in totals as included above
EBSCO	“physical activity” OR exercise OR fitness OR “physical exercise” OR diet OR nutrition *n =* 247,227	“young adult*” OR “emerging adult*” OR “young people” *n =* 8355	influence* OR “social influences” OR peer OR parent OR partner OR environment OR work* OR school OR university OR “social media” OR advertising OR “health communication” OR “video game*” OR “online game*” OR “computer game*” OR government OR “social norm” OR celebrity OR microcelebrity OR “physical activity environment” OR obesogenic OR “food environment”	2010–2020	3801	
EBSCO	“physical activity” OR exercise OR fitness OR “physical exercise” OR diet OR nutrition *n =* 247,227	“young adult*” OR “emerging adult*” OR “young people” *n =* 8355	“video game*” OR “online game*” OR “computer game*”	2010–2020	70	*not counted in totals as included above
Google Scholar	“physical activity” OR exercise OR fitness OR “physical exercise” OR diet OR nutrition *n =* 676,000	“young adult*” OR “emerging adult*” OR “young people” *n =* 23,500	influence* OR “social influences” OR peer OR parent OR partner OR environment OR work* OR school	2010–2020	18,600	
Google Scholar	“physical activity” OR exercise OR fitness OR “physical exercise” OR diet OR nutrition *n =* 676,000	“young adult*” OR “emerging adult*” OR “young people” *n =* 23,500	university OR “social media” OR advertising OR “health communication”	2010–2020	19,200	
Google Scholar	“physical activity” OR exercise OR fitness OR “physical exercise” OR diet OR nutrition *n =* 676,000	“young adult*” OR “emerging adult*” OR “young people” *n =* 23,500	video game* OR “online game*” OR “computer game*”	2010–2020	17,100	
Google Scholar	“physical activity“OR exercise OR fitness OR “physical exercise” OR diet OR nutrition *n =* 676,000	“young adult*” OR “emerging adult*” OR “young people” *n =* 23,500	government OR “social norm” OR celebrity OR microcelebrity OR “physical activity environment” OR obesogenic OR “food environment”	2010–2020	20,100	
**Totals ***						
Proquest	67,649					
EBSCO	3801					
Google Scholar	75,000					

* Searches were conducted in December 2020 and total number may vary in consecutive searches.
