# Supplementary material for: Where Do Online Games Fit into the Health Behaviour Ecology of Emerging Adults: A Scoping Review
Source: Nutrients. 2021 Aug 23;13(8):2895. doi: 10.3390/nu13082895 (PMC8400136; doi:10.3390/nu13082895)
Supplement: Supplementary file 1 [file nutrients-13-02895-s001.zip › nutrients-1292840-supplementary.pdf]

Supplementary Reference List – articles included in scoping review

1. Adachi, P.J.; Willoughby, T. Does Playing Sports Video Games Predict Increased Involvement in Real-Life Sports Over Several Years Among Older Adolescents and Emerging Adults? *Journal of Youth and Adolescence* **2016**, *45*, 391-401, doi:http://dx.doi.org/10.1007/s10964-015-0312-2.
2. Adachi, P.J.; Willoughby, T. The Link Between Playing Video Games and Positive Youth Outcomes. *Child Development Perspectives* **2017**, *11*, 202-206, doi:10.1111/cdep.12232.
3. Al-Eisa, E.; Al-Rushud, A.; Alghadir, A.; Anwer, S.; Al-Harbi, B.; Al-Sughaier, N.; Al-Yoseef, N.; Al-Otaibi, R.; Al-Muhaysin, H.A. Effect of Motivation by "Instagram" on Adherence to Physical Activity among Female College Students. *BioMed Research International* **2016**, *2016*, 1546013, doi:10.1155/2016/1546013.
4. Allom, V.; Mullan, B. Maintaining healthy eating behaviour: experiences and perceptions of young adults. *Nutrition and food science* **2014**, *44*, 156-167, doi:10.1108/nfs-06-2013-0077.
5. Allom, V.; Mullan, B.; Cowie, E.; Hamilton, K. Physical Activity and Transitioning to College: The Importance of Intentions and Habits. *American Journal of Health Behavior* **2016**, *40*, 280-290, doi:10.5993/AJHB.40.2.13.
6. Amuta, A.O.; Jacobs, W.; Barry, A.E. An Examination of Family, Healthcare Professionals, and Peer Advice on Physical Activity Behaviors among Adolescents at High Risk for Type 2 Diabetes. *Health Communication* **2017**, *32*, 857-863, doi:10.1080/10410236.2016.1177907.
7. Ashton, L.M.; Hutchesson, M.J.; Rollo, M.E.; Morgan, P.J.; Thompson, D.I.; Collins, C.E. Young adult males' motivators and perceived barriers towards eating healthily and being active: a qualitative study. *International Journal of Behavioral Nutrition and Physical Activity* **2015**, *12*, 93, doi:10.1186/s12966-015-0257-6.
8. Baiocchi-Wagner, E.A.; Talley, A.E. The Role of Family Communication in Individual Health Attitudes and Behaviors Concerning Diet and Physical Activity. *Health Commun* **2013**, *28*, 193-205, doi:10.1080/10410236.2012.674911.
9. Ball, K.; Jeffery, R.W.; Abbott, G.; McNaughton, S.A.; Crawford, D. Is healthy behavior contagious: associations of social norms with physical activity and healthy eating. *Int J Behav Nutr Phys Act* **2010**, *7*, 86-86, doi:10.1186/1479-5868-7-86.
10. Barklamb, A.M.; Molenaar, A.; Brennan, L.; Evans, S.; Choong, J.; Herron, E.; Reid, M.; McCaffrey, T. Learning the Language of Social media: A Comparison of Engagement Metrics and Social Media Strategies Used by Food and Nutrition-Related Social Media Accounts. *Nutrients* **2020**, *12*, doi:https://doi.org/10.3390/nu12092839.
11. Barnett, N.P.; Ott, M.Q.; Rogers, M.L.; Loxley, M.; Linkletter, C.; Clark, M.A. Peer associations for substance use and exercise in a college student social network. *Health Psychology* **2014**, *33*, 1134-1142, doi:http://dx.doi.org/10.1037/a0034687.
12. Belanger, N.M.S.; Patrick, J.H. The Influence of Source and Type of Support on College Students' Physical Activity Behavior. *J Phys Act Health* **2018**, *15*, 183-190, doi:10.1123/jpah.2017-0069.
13. Bell, S.; Lee, C. Emerging Adulthood and Patterns of Physical Activity Among Young Australian Women. *International Journal of Behavioral Medicine* **2005**, *12*, 227-235.
14. Berg, C.J.; An, L.C.; Ahluwalia, J.S. Dietary Fat Intake and Exercise Among Two- and Four-Year College Students: Differences in Behavior and Psychosocial Factors. *Community College Journal of Research and Practice* **2013**, *37*, 388-396, doi:10.1080/10668921003609285.
15. Berry, E.; Aucott, L.; Poobalan, A. Are young adults appreciating the health promotion messages on diet and exercise? *Journal of Public Health* **2018**, *26*, 687-696, doi:http://dx.doi.org/10.1007/s10389-018-0905-9.
16. Berzins, T.L.; Gere, J.; Kelly, S.M.; Updegraff, J.A. Associations between social control, motivation, and exercise: How romantic partners influence exercise during young adulthood. *J Health Psychol* **2017**, *24*, 1425-1435, doi:10.1177/1359105317727840.
17. Bray, S.R.; Born, H.A. Transition to University and Vigorous Physical Activity: Implications for Health and Psychological Well-Being. *Journal of American College Health* **2004**, *52*, 181-188, doi:10.3200/JACH.52.4.181-188.
18. Brennan, L.; Klassen, K.; Weng, E.; Chin, S.; Molenaar, A.; Reid, M.; Truby, H.; McCaffrey, T.A. A social marketing perspective of young adults' concepts of eating for health: is it a question of morality? *International Journal of Behavioral Nutrition and Physical Activity* **2020**, *17*, doi:10.1186/s12966-020-00946-3.
19. Brennan, L.; Shinyi, C.; Annika, M.; Amy, M.B.; Megan, S.C.L.; Mike, R.; Helen, T.; Eva, L.J.; Tracy, A.M. Beyond Body Weight: Design and Validation of Psycho-Behavioural Living and Eating for Health Segments (LEHS) Profiles for Social Marketing. *Nutrients* **2020**, *12*, 2882, doi:10.3390/nu12092882.

20. Brown, D.M.Y.; Bray, S.R.; Beatty, K.R.; Kwan, M.Y.W. Healthy Active Living: A Residence Community–Based Intervention to Increase Physical Activity and Healthy Eating During the Transition to First-Year University. *Journal of American College Health* **2014**, *62*, 234-242, doi:10.1080/07448481.2014.887572.
21. Bruening, M.; Huberty, J.; Skelton, K.; Brennhofer, S.; Voytyuk, M. Social Mechanisms for Weight-related Behaviors among Emerging Adults. *Health behavior and policy review* **2017**, *4*, 419-426, doi:http://dx.doi.org/10.14485/HBPR.4.5.1.
22. Budzynski-Seymour, E.; Conway, R.; Wade, M.; Lucas, A.; Jones, M.; Mann, S.; Steele, J. Physical Activity, Mental and Personal Well-Being, Social Isolation, and Perceptions of Academic Attainment and Employability in University Students: The Scottish and British Active Students Surveys. *Journal of Physical Activity and Health* **2020**, *17*, 610-620, doi:10.1123/jpah.2019-0431.
23. Burgess, E.R.; Walter, N.; Ball-Rokeach, S.J.; Murphy, S.T. Communication Hotspots: How Infrastructure Shapes People's Health. *Health Communication* **2019**, 10.1080/10410236.2019.1692490, doi:10.1080/10410236.2019.1692490.
24. Burns, S.; Evans, M.; Jancey, J.; Portsmouth, L.; Maycock, B. Influences of club connectedness among young adults in Western Australian community-based sports clubs. *BMC Public Health* **2020**, *20*, 1-14, doi:http://dx.doi.org/10.1186/s12889-020-08836-w.
25. Carballo-Fazanes, A.; Rico-Díaz, J.; Barcala-Furelos, R.; Rey, E.; Rodríguez-Fernández, J.E.; Varela-Casal, C.; Abelairas-Gómez, C. Physical Activity Habits and Determinants, Sedentary Behaviour and Lifestyle in University Students. *International Journal of Environmental Research and Public Health* **2020**, *17*, 3272, doi:http://dx.doi.org/10.3390/ijerph17093272.
26. Chacón Cuberos, R.; Zurita Ortega, F.; Puertas Molero, P.; Knox, E.; Cofré Bolados, C.; Viciana Garófano, V.; Muros Molina, J.J. Relationship between Healthy Habits and Perceived Motivational Climate in Sport among University Students: A Structural Equation Model. *Sustainability* **2018**, *10*, 938.
27. Chan, G.; Arya, A.; Orji, R.; Zhao, Z. Motivational strategies and approaches for single and multi-player exergames: a social perspective. *PeerJ Computer Science* **2019**, http://dx.doi.org/10.7717/peerj-cs.230, doi:http://dx.doi.org/10.7717/peerj-cs.230.
28. Chim, H.Q.; oude Egbrink, M.G.A.; Van Gerven, P.W.M.; de Groot, R.H.M.; Winkens, B.; Savelberg, H.H.C.M. Academic Schedule and Day-to-Day Variations in Sedentary Behavior and Physical Activity of University Students. *International Journal of Environmental Research and Public Health* **2020**, *17*, 2810, doi:http://dx.doi.org/10.3390/ijerph17082810.
29. Chwialkowska, A. The Role of the Family in the Adoption of a Vegan Diet. The Implications for Consumer Socialization towards Sustainable Food Consumption. *Journal of Marketing Development & Competitiveness* **2018**, *12*, 11-37, doi:10.33423/jmdc.v12i4.196.
30. Corder, K.; Winpenny, E.; Love, R.; Brown, H.E.; White, M.; Sluijs, E.v. Change in physical activity from adolescence to early adulthood: a systematic review and meta-analysis of longitudinal cohort studies. *British Journal of Sports Medicine* **2019**, *53*, 496-503, doi:10.1136/bjsports-2016-097330.
31. Diehl, K.; Hilger, J. Physical activity and the transition from school to university: A cross-sectional survey among university students in Germany. *Science & Sports* **2016**, *31*, 223-226, doi:https://doi.org/10.1016/j.scispo.2016.04.012.
32. Divine, A.; Watson, P.M.; Baker, S.; Hall, C.R. Facebook, relatedness and exercise motivation in university students: A mixed methods investigation. *Computers in Human Behavior* **2019**, *91*, 138-150, doi:http://dx.doi.org/10.1016/j.chb.2018.09.037.
33. Draper, C.E.; Grobler, L.; Micklesfield, L.K.; Norris, S.A. Impact of social norms and social support on diet, physical activity and sedentary behaviour of adolescents: a scoping review. *Child: Care Health & Development* **2015**, *41*, 654-667.
34. Fergie, G.; Hunt, K.; Hilton, S. What young people want from health-related online resources: a focus group study. *J Youth Stud* **2013**, *16*, 579-596, doi:10.1080/13676261.2012.744811.
35. Fletcher, A.; Bonell, C.; Sorhaindo, A. You are what your friends eat: systematic review of social network analyses of young people's eating behaviours and bodyweight. *J Epidemiol Community Health* **2011**, *65*, 548-555, doi:10.1136/jech.2010.113936.
36. Freeman, B.; Kelly, B.; Vandevijvere, S.; Baur, L. Young adults: beloved by food and drink marketers and forgotten by public health? *Health Promot Int* **2016**, *31*, 954.
37. Gallacher-Parousis, F. Exploring the Factors Influencing Emerging Adults Decision to Engage in Physical Activity. Ph.D., Northcentral University, Ann Arbor, 2018.
38. Giles, E.L.; Brennan, M. Trading between healthy food, alcohol and physical activity behaviours. *BMC Public Health* **2014**, *14*, 1231-1231, doi:10.1186/1471-2458-14-1231.

39. Goodyear, V.A.; Armour, K.M. Young People's Perspectives on and Experiences of Health-Related Social Media, Apps, and Wearable Health Devices. *Social Sciences* **2018**, *7*, doi:http://dx.doi.org/10.3390/socsci7080137.
40. Goodyear, V.A.; Armour, K.M.; Wood, H. Young people and their engagement with health-related social media: new perspectives. *Sport, Education and Society* **2019**, *24*, 673-688, doi:10.1080/13573322.2017.1423464.
41. Graham, D.J.; Pelletier, J.E.; Neumark-Sztainer, D.; Lust, K.; Laska, M.N. Perceived Social-Ecological Factors Associated with Fruit and Vegetable Purchasing, Preparation, and Consumption among Young Adults. *Journal of the Academy of Nutrition and Dietetics* **2013**, *113*, 1366-1374, doi:https://doi.org/10.1016/j.jand.2013.06.348.
42. Gropper, H.; John, J.M.; Sudeck, G.; Thiel, A. The impact of life events and transitions on physical activity: A scoping review. *PLOS ONE* **2020**, *15*, e0234794, doi:10.1371/journal.pone.0234794.
43. Guntzviller, L.M.; Ratcliff, C.L.; Dorsch, T.E.; Osai, K.V. How do emerging adults respond to exercise advice from parents? A test of advice response theory. *Journal of Social and Personal Relationships* **2017**, *34*, 936-960, doi:10.1177/0265407516662920.
44. Hafiz, A.A.; Gallagher, A.M.; Hill, A.J. The influence of social networks in increasing fruit and vegetables consumption in university students: a randomised controlled study. *Proc. Nutr. Soc* **2018**, *77*, doi:10.1017/S0029665117004384.
45. Hamari, J.; Koivisto, J. "Working out for likes": An empirical study on social influence in exercise gamification. *Computers in human behavior* **2015**, *50*, 333-347, doi:10.1016/j.chb.2015.04.018.
46. Harmon, B.E.; Forthofer, M.; Bantum, E.O.; Nigg, C.R. Perceived influence and college students' diet and physical activity behaviors: an examination of ego-centric social networks. *BMC Public Health* **2016**, *16*, 473-410, doi:10.1186/s12889-016-3166-y.
47. Higgs, S.; Thomas, J. Social influences on eating. *Current Opinion in Behavioral Sciences* **2016**, *9*, 1-6, doi:https://doi.org/10.1016/j.cobeha.2015.10.005.
48. Holley, T.J.; Collins, C.E.; Morgan, P.J.; Callister, R.; Hutchesson, M.J. Weight expectations, motivations for weight change and perceived factors influencing weight management in young Australian women: a cross-sectional study. *Public Health Nutr* **2016**, *19*, 275-286, doi:10.1017/S1368980015000993.
49. Holt-Lunstad, J. Why Social Relationships Are Important for Physical Health: A Systems Approach to Understanding and Modifying Risk and Protection. *Annual Review of Psychology* **2018**, *69*, 437-458, doi:10.1146/annurev-psych-122216-011902.
50. Hong, Y.; Kim, S. Influence of Presumed Media Influence for Health Prevention: How Mass Media Indirectly Promote Health Prevention Behaviors through Descriptive Norms. *Health Commun* **2019**, 10.1080/10410236.2019.1663585, 1-11, doi:10.1080/10410236.2019.1663585.
51. Jenkins, E.L.; Ilicic, J.; Barklamb, A.M.; McCaffrey, T.A. Assessing the Credibility and Authenticity of Social Media Content for Applications in Health Communication: Scoping Review. *Journal of medical Internet research* **2020**, *22*, e17296, doi:10.2196/17296.
52. Kapinos, K.A.; Yakusheva, O.; Eisenberg, D. Obesogenic environmental influences on young adults: Evidence from college dormitory assignments. *Econ Hum Biol* **2014**, *12*, 98-109, doi:10.1016/j.ehb.2013.05.003.
53. Kim, G.S.; Lee, C.Y.; Kim, I.S.; Lee, T.H.; Cho, E.; Lee, H.; McCreary, L.L.; Kim, S.H. Dyadic Effects of Individual and Friend on Physical Activity in College Students. *Public Health Nurs* **2015**, *32*, 430-439, doi:10.1111/phn.12176.
54. Kinard, B.R.; Webster, C. Factors influencing unhealthy eating behaviour in US adolescents. *International Journal of Consumer Studies* **2012**, *36*, 23-29, doi:10.1111/j.1470-6431.2011.01005.x.
55. King, K.A.; Vidourek, R.A.; English, L.; Merianos, A.L. Vigorous physical activity among college students: Using the health belief model to assess involvement and social support. *Archives of exercise in health and disease* **2014**, *4*, 267-279, doi:10.5628/aehd.v4i2.153.
56. Klaiber, P.; Whillans, A.V.; Chen, F.S. Long-Term Health Implications of Students' Friendship Formation during the Transition to University. *Appl Psychol Health Well Being* **2018**, *10*, 290-308, doi:10.1111/aphw.12131.
57. Klassen, K.M.; Douglass, C.H.; Brennan, L.; Truby, H.; Lim, M.S.C. Social media use for nutrition outcomes in young adults: a mixed-methods systematic review. *Int J Behav Nutr Phys Act* **2018**, *15*, 70-18, doi:10.1186/s12966-018-0696-y.
58. Koivisto, J.; Malik, A.; Gurer Gurkan, B.; Hamari, J. Getting Healthy by Catching Them All: A Study on the Relationship between Player Orientations and Perceived Health Benefits in an Augmented Reality Game. In Proceedings of 52nd Hawaii International Conference on System Sciences, Hawaii, US; pp. 1779 - 1788.
59. König, L.M.; Giese, H.; Stok, F.M.; Renner, B. The social image of food: Associations between popularity and eating behavior. *Appetite* **2017**, *114*, 248-258, doi:10.1016/j.appet.2017.03.039.

60. Kwan, M.Y.; Cairney, J.; Faulkner, G.E.; Pullenayegum, E.E. Physical activity and other health-risk behaviors during the transition into early adulthood: a longitudinal cohort study. *Am J Prev Med* **2012**, *42*, 14.
61. LaChausse, R.G. My Student Body: Effects of an Internet-Based Prevention Program to Decrease Obesity Among College Students. *J Am Coll Health* **2012**, *60*, 324-330, doi:10.1080/07448481.2011.623333.
62. Lambert, M.; Chivers, P.; Farringdon, F. In their own words: A qualitative study exploring influences on the food choices of university students. *Health Promot J Austr* **2019**, *30*, 66-75, doi:10.1002/hpja.180.
63. Langdon, J.; Johnson, C.; Melton, B. Factors contributing to the uptake and maintenance of regular exercise behaviour in emerging adults. *The Health Education Journal* **2017**, *76*, 182-193, doi:http://dx.doi.org/10.1177/0017896916654934.
64. Larson, N.; Chen, Y.; Wall, M.; Winkler, M.R.; Goldschmidt, A.B.; Neumark-Sztainer, D. Personal, behavioral, and environmental predictors of healthy weight maintenance during the transition to adulthood. *Prev Med* **2018**, *113*, 80-90, doi:10.1016/j.ypmed.2018.04.027.
65. Larson, N.; Laska, M.N.; Story, M.; Neumark-Sztainer, D. Predictors of Fruit and Vegetable Intake in Young Adulthood. *J Acad Nutr Diet* **2012**, *112*, 1216-1222, doi:10.1016/j.jand.2012.03.035.
66. Laska, M.N.; Sevcik, S.M.; Moe, S.G.; Petrich, C.A.; Nanney, M.S.; Linde, J.A.; Lytle, L.A. A 2-year young adult obesity prevention trial in the US: Process evaluation results. *Health Promot Int* **2016**, *31*, 793-800, doi:10.1093/heapro/dav066.
67. Laska, M.N.; VanKim, N.A.; Erickson, D.J.; Lust, K.; Eisenberg, M.E.; Rosser, B.R.S. Disparities in Weight and Weight Behaviors by Sexual Orientation in College Students. *American Journal of Public Health* **2015**, *105*, 111-121.
68. Leslie, E.; Owen, N.; Salmon, J.; Bauman, A.; Sallis, J.F.; Lo, S.K. Insufficiently Active Australian College Students: Perceived Personal, Social, and Environmental Influences. *Prev Med* **1999**, *28*, 20-27, doi:10.1006/pmed.1998.0375.
69. Levinger, P.; Hill, K.D. The Impact of Mass Media Campaigns on Physical Activity Participation on a Global Scale: Lessons Learned From the COVID-19 Pandemic. *Journal of Physical Activity and Health* **2020**, 10.1123/jpah.2020-0387, doi:10.1123/jpah.2020-0387.
70. Li, B.; Lwin, M. Player see, player do: Testing an exergame motivation model based on the influence of the self avatar. *Computers in Human Behavior* **2016**, *59*, 350-357, doi:https://doi.org/10.1016/j.chb.2016.02.034.
71. Li, K.; Liu, D.; Haynie, D.; Gee, B.; Chaurasia, A.; Seo, D.-C.; Iannotti, R.J.; Simons-Morton, B.G. Individual, social, and environmental influences on the transitions in physical activity among emerging adults. *BMC Public Health* **2016**, *16*, 682, doi:10.1186/s12889-016-3368-3.
72. Lilienthal, K.R.; Holm, J.; Vogeltanz-Holm, N. The influence of friend presence and discussion on young adults' responses to anti-sugary drinks television ads. *Current Psychology: A Journal for Diverse Perspectives on Diverse Psychological Issues* **2015**, *34*, 401-421, doi:http://dx.doi.org/10.1007/s12144-014-9265-y.
73. Lindgren, E.-C.; Annerstedt, C.; Dohsten, J. "The individual at the centre" – a grounded theory explaining how sport clubs retain young adults. *International Journal of Qualitative Studies on Health and Well-Being* **2017**, *12*, 1-12, doi:http://dx.doi.org/10.1080/17482631.2017.1361782.
74. Lippert, A.M. Stuck in Unhealthy Places: How Entering, Exiting, and Remaining in Poor and Nonpoor Neighborhoods Is Associated with Obesity during the Transition to Adulthood. *J Health Soc Behav* **2016**, *57*, 1-21, doi:10.1177/0022146515627682.
75. Lupton, D. 'Better Understanding about What's Going On': Young Australians' Use of Digital Technologies for Health and Fitness. *Sport, Education and Society* **2020**, *25*, 1-13, doi:http://dx.doi.org/10.1080/13573322.2018.1555661.
76. Magoc, D.; Tomaka, J.; Bridges-Arzaga, A. Using the web to increase physical activity in college students. *Am J Health Behav* **2011**, *35*, 142-154, doi:10.5993/AJHB.35.2.2.
77. Mann, L.; Blotnicky, K. University Students' Eating Behaviors: An Exploration of Influencers. *College Student Journal* **2016**, *50*, 489-500.
78. Marquis, M.; Talbot, A.; Sabourin, A.; Riopel, C. Exploring the environmental, personal and behavioural factors as determinants for university students' food behaviour. *International Journal of Consumer Studies* **2019**, *43*, 113-122, doi:10.1111/ijcs.12490.
79. Meng, J.; Peng, W.; Shin, S.Y.; Chung, M. Online Self-Tracking Groups to Increase Fruit and Vegetable Intake: A Small-Scale Study on Mechanisms of Group Effect on Behavior Change. *J Med Internet Res* **2017**, *19*, e63-e63, doi:10.2196/jmir.6537.
80. Miller, C.; Braunack-Mayer, A.; Wakefield, M.; Roder, D.; O'Dea, K.; Dono, J.; Ettridge, K. " When we were young, it really was a treat; now sugar is just the norm every day " — A qualitative study of parents' and young adults' perceptions and consumption of sugary drinks. *Health Promotion Journal of Australia* **2020**, *31*, 47–57, doi:10.1002/hpja.257.

81. Molenaar, A.; Choi, T.S.; Brennan, L.; Reid, M.; Lim, M.S.; Truby, H.; McCaffrey, T.A. Language of Health of Young Australian Adults: A Qualitative Exploration of Perceptions of Health, Wellbeing and Health Promotion via Online Conversations. *Nutrients* **2020**, *12*, 887, doi:10.3390/nu12040887.
82. Munt, A.E.; Partridge, S.R.; Allman-Farinelli, M. The barriers and enablers of healthy eating among young adults: a missing piece of the obesity puzzle: A scoping review: Barriers and enablers of healthy eating. *Obesity reviews* **2017**, *18*, 1-17, doi:10.1111/obr.12472.
83. Navarro, J.; Peña, J.; Cebolla, A.; Baños, R. Can Avatar Appearance Influence Physical Activity? User-Avatar Similarity and Proteus Effects on Cardiac Frequency and Step Counts. *Health Communication* **2020**, 10.1080/10410236.2020.1834194, doi:10.1080/10410236.2020.1834194.
84. Nour, M.; Sui, Z.; Grech, A.; Rangan, A.; McGeechan, K.; Allman-Farinelli, M. The fruit and vegetable intake of young Australian adults: a population perspective. *Public Health Nutr* **2017**, *20*, 2499-2512, doi:10.1017/S1368980017001124.
85. Otundo, J.O.; MacGregor, S.K. Effect of Situational Interest and Social Support on College Students' Physical Activity Motivation: A Mixed Methods Analysis. *Physical Educator* **2019**, *76*, 502-523, doi:http://dx.doi.org/10.18666/TPE-2019-V76-I2-8502.
86. Paluch, A.E.; Shook, R.P.; Hand, G.A.; O'Connor, D.P.; Wilcox, S.; Drenowatz, C.; Baruth, M.; Burgess, S.; Blair, S.N. The Influence of Life Events and Psychological Stress on Objectively Measured Physical Activity: A 12-Month Longitudinal Study. *Journal of Physical Activity & Health* **2018**, *15*, 374-382.
87. Pelletier, J.E.; Graham, D.J.; Laska, M.N. Social norms and dietary behaviors among young adults. *Am J Health Behav* **2014**, *38*, 144-152, doi:10.5993/AJHB.38.1.15.
88. Pelletier, J.E.; Laska, M.N. Balancing Healthy Meals and Busy Lives: Associations between Work, School, and Family Responsibilities and Perceived Time Constraints among Young Adults. *J Nutr Educ Behav* **2012**, *44*, 481-489, doi:10.1016/j.jneb.2012.04.001.
89. Piggford, T.; Raciti, M.; Harker, D.; Harker, M. Young adults' food motives: an Australian social marketing perspective. *Young Consumers* **2008**, *9*, 17-28, doi:10.1108/17473610810857282.
90. Plotnikoff, R.C.; Costigan, S.A.; Williams, R.L.; Hutchesson, M.J.; Kennedy, S.G.; Robards, S.L.; Allen, J.; Collins, C.E.; Callister, R.; Germov, J. Effectiveness of interventions targeting physical activity, nutrition and healthy weight for university and college students: a systematic review and meta-analysis. *International Journal of Behavioral Nutrition and Physical Activity* **2015**, *12*, 45, doi:10.1186/s12966-015-0203-7.
91. Poobalan, A.S.; Aucott, L.S.; Clarke, A.; Smith, W.C.S. Physical activity attitudes, intentions and behaviour among 18–25 year olds: A mixed method study. *BMC Public Health* **2012**, *12*, 640, doi:10.1186/1471-2458-12-640.
92. Pugliese, J.A.; Okun, M.A. Social control and strenuous exercise among late adolescent college students: Parents versus peers as influence agents. *Journal of Adolescence* **2014**, *37*, 543-554, doi:https://doi.org/10.1016/j.adolescence.2014.04.008.
93. Robinson, E. Perceived social norms and eating behaviour: An evaluation of studies and future directions. *Physiology & Behavior* **2015**, *152*, 397-401, doi:https://doi.org/10.1016/j.physbeh.2015.06.010.
94. Robinson, E.; Tobias, T.; Shaw, L.; Freeman, E.; Higgs, S. Social matching of food intake and the need for social acceptance. *Appetite* **2011**, *56*, 747-752, doi:10.1016/j.appet.2011.03.001.
95. Rounsefell, K.; Gibson, S.; McLean, S.; Blair, M.; Molenaar, A.; Brennan, L.; Truby, H.; McCaffrey, T.A. Social media, body image and food choices in healthy young adults: A mixed methods systematic review. *Nutr Diet* **2019**, *77*, 19-40, doi:10.1111/1747-0080.12581.
96. Sbaffi, L.; Zhao, C. Modeling the online health information seeking process: Information channel selection among university students. *Journal of the Association for Information Science and Technology* **2020**, *71*, 196-207, doi:10.1002/asi.24230.
97. Scarapicchia, T.M.F.; Sabiston, C.M.; Pila, E.; Arbour-Nicitopoulos, K.P.; Faulkner, G. A longitudinal investigation of a multidimensional model of social support and physical activity over the first year of university. *Psychology of Sport and Exercise* **2017**, *31*, 11-20, doi:http://dx.doi.org/10.1016/j.psychsport.2017.03.011.
98. Sharps, M.A.; Hetherington, M.M.; Blundell-Birtill, P.; Rolls, B.J.; Evans, C.E.L. The effectiveness of a social media intervention for reducing portion sizes in young adults and adolescents. *Digital Health* **2019**, *5*, doi:http://dx.doi.org/10.1177/2055207619878076.
99. Sogari, G.; Velez-Argumedo, C.; Gómez, M.; Mora, C. College Students and Eating Habits: A Study Using An Ecological Model for Healthy Behavior. *Nutrients* **2018**, *10*, 1823, doi:10.3390/nu10121823.

100. Sullivan, K.T.; Pasch, L.A.; Schreier, M.; Healy, M. Responses to intimate partners' attempts to change health behavior: The role of readiness. *Journal of Social and Personal Relationships* **2018**, *35*, 1356-1380, doi:10.1177/0265407517713364.
101. Szuhany, K.L.; Otto, M.W. The new TV dinner: effects of television programming content on eating and attitudes towards exercise. *Psychol Health Med* **2019**, *25*, 1-6, doi:10.1080/13548506.2019.1705990.
102. Townshend, T.; Lake, A. Obesogenic environments: current evidence of the built and food environments. *Perspectives in Public Health* **2017**, *137*, 38-44, doi:http://dx.doi.org/10.1177/1757913916679860.
103. Uijtdewilligen, L.; Peeters, G.M.E.E.; van Uffelen, J.G.Z.; Twisk, J.W.R.; Singh, A.S.; Brown, W.J. Determinants of physical activity in a cohort of young adult women. Who is at risk of inactive behaviour? *Journal of Science & Medicine in Sport* **2015**, *18*, 49-55.
104. van Houten, J.M.A.; Kraaykamp, G.; Pelzer, B.J. The transition to adulthood: a game changer!? A longitudinal analysis of the impact of five major life events on sport participation. *European Journal for Sport and Society* **2019**, *16*, 44-63, doi:10.1080/16138171.2019.1603832.
105. VanKim, N.A.; Porta, C.M.; Eisenberg, M.E.; Neumark-Sztainer, D.; Laska, M.N. Lesbian, gay and bisexual college student perspectives on disparities in weight-related behaviours and body image: a qualitative analysis. *J Clin Nurs* **2016**, *25*, 3676-3686, doi:10.1111/jocn.13106.
106. Vaterlaus, J.M.; Patten, E.V.; Roche, C.; Young, J.A. #Gettinghealthy: The perceived influence of social media on young adult health behaviors. *Computers in Human Behavior* **2015**, *45*, 151-157, doi:https://doi.org/10.1016/j.chb.2014.12.013.
107. Walker, T.; Molenaar, A.; Palermo, C. A qualitative study exploring what it means to be healthy for young Indigenous Australians and the role of social media in influencing health behaviour. *Health Promotion Journal of Australia* **2020**, 10.1002/hpja.391, doi:10.1002/hpja.391.
108. Walsh, A.; Taylor, C.; Brennick, D. Factors That Influence Campus Dwelling University Students' Facility to Practice Healthy Living Guidelines. *Can J Nurs Res* **2018**, *50*, 57-63, doi:10.1177/0844562117747434.
109. Watts, A.W.; Laska, M.N.; Larson, N.I.; Neumark-Sztainer, D.R. Millennials at work: workplace environments of young adults and associations with weight-related health. *Journal of Epidemiology & Community Health* **2016**, *70*, 65-71.
110. Wong, F.Y. Influence of Pokémon Go on physical activity levels of university players: a cross-sectional study. *Int J Health Geogr* **2017**, *16*, 8, doi:10.1186/s12942-017-0080-1.
111. Yoon, A.; Choi, S.; Mun, J.; Hong, J.; Hahn, D.; Kang, M.; Lee, S. Motivational signage increases stair usage on a Hispanic serving institution. *Journal of American College Health* **2020**, *68*, 236–241, doi:10.1080/07448481.2018.1539000.
112. Zhan, C.; Heatherington, L.; Klingenberg, B. Disordered eating- and exercise-related behaviors and cognitions during the first year college transition. *Journal of American College Health* **2020**, 10.1080/07448481.2020.1775608, doi:10.1080/07448481.2020.1775608.
